# Metallic lead (Pb) nanospheres discovered in Hadean and Eoarchean zircon crystals at Jack Hills

**DOI:** 10.1038/s41598-023-27843-6

**Published:** 2023-01-17

**Authors:** Monika A. Kusiak, Richard Wirth, Simon A. Wilde, Robert T. Pidgeon

**Affiliations:** 1 Institute of Geophysics, Polish Academy of Science, ul. Księcia Janusza 64, 01452 Warsaw, Poland; 2 Section 3.5 Surface Geochemistry, GeoForschungsZentrum, Potsdam, Telegrafenberg, 14473 Potsdam, Germany; 3 School of Earth and Planetary Sciences, Curtin University, PO Box U1987, Perth, WA 6845 Australia

**Keywords:** Planetary science, Solid Earth sciences

## Abstract

Here, we report small randomly-distributed crystalline lead (Pb) nanospheres occurring in detrital zircon grains obtained from a weakly metamorphosed Archean conglomerate at Jack Hills, Western Australia, making this the third known global example of this phenomenon. They form in zircon crystals ranging from Hadean (> 4 billion years—Ga) to Eoarchean (> 3.6 Ga) in age, but are absent from Paleoarchean (~ 3.4 Ga) crystals. Unlike previous discoveries of nanospheres in zircon from Precambrian gneisses in Antarctica and India, detrital zircon from Jack Hills shows no evidence of ever undergoing ultra-high temperature (UHT) metamorphism, either before or after deposition, therefore implying that nanospheres can form at temperatures lower than ca. 900 °C. The nanospheres are composed of radiogenic Pb released by the breakdown of uranium (U) and thorium (Th) and are present in zircon irrespective of its U, Th and water contents, its oxygen isotopic composition, and the degree of discordance due to Pb loss or gain. The nanospheres pre-date annealed cracks in the crystals, showing that, once formed, they effectively ‘freeze’ radiogenic Pb in the zircon structure, precluding any further interaction during subsequent geological processes. Both Pb nanoclusters and nanospheres are now reported from Jack Hills, and it appears likely the former is a precursor stage in the formation of the latter. Although the precise mechanism for this transition remains unresolved, a later thermal event is required, but this likely did not reach UHT conditions at Jack Hills.

## Introduction

Uranium-lead dating of zircon (ZrSiO_4_) is considered the best geochronometer for determining events on the early Earth, because a basic tenet is that Pb is generally not incorporated into the zircon structure during growth. The reason for this is evident from the three possible ionic radii of lead: Pb^0^ (2.02 Å), Pb^2+^ (1.29 Å) and Pb^4+^ (0.94 Å), as only the latter has an ionic radius and charge that would allow substitution for Zr^4+^ (0.84 Å), although extremely oxidizing conditions would be required to keep it stable in the zircon structure^1,2^. Nevertheless, there is no agreement as to the oxidation state of Pb in zircon, with some considering it tetravalent^3^, others divalent^4^, or even a mixture of the two^5^. Lead can also be concentrated locally along cleavage planes, occur as Pb-rich particles associated with pores, form Pb phosphate, and form Pb-oxide nanoparticles associated with dislocations^2,6,7^. The lower the degree of compatibility of Pb in zircon, the greater the diffusion rate^8^, particularly within amorphous domains created by radiation damage resulting from the decay of U and Th to radiogenic lead (Pb*). Similarly, progressive thermal annealing^9^ and fluid infiltration^10^ during metamorphism can also result in Pb* mobility. However, assigning specific processes to individual examples is difficult^11^.

Recent studies have revealed further complications that could potentially question the reliability of zircon geochronological data, showing it can be compromised by re-mobilization of Pb* at the micro- and nano-scales^12–16^. This has been shown to occur in zircon at very high temperature and/or pressure, and is particularly significant for old grains with complex histories, where multiple dates may be recorded within a single crystal^15^.

The first evidence of Pb* mobility in zircon was observed in Archean orthogneisses from the Napier Complex in East Antarctica^17^, where it was proposed that within-run variations in Pb content during analysis by secondary-ion mass spectrometry (SIMS), and the resultant reversely discordant data (^206^Pb/^238^U > ^207^Pb/^206^Pb ages), were the result of excess Pb*. It was later shown^18^ that Pb* in zircon from the same field sites in Antarctica was concentrated in loose nanoclusters, considered to have formed during ultra-high temperature (UHT) metamorphism, where temperatures exceeded 1120 °C @ ~ 2.5 Ga^19^. Further research on these samples led to the eventual discovery of metallic Pb nanospheres^20^, which have since been documented in zircon from similar UHT gneisses of Paleoproterozoic age in the Kerala Khondalite Belt of southern India^21^.

It is important here to stress the difference between Pb nanoclusters and Pb nanospheres, as the distinction is not always appreciated. Lead nanoclusters are loose concentrations of Pb* atoms, whereas Pb nanospheres are actual crystals composed of metallic Pb with a cubic symmetry^20^. Several studies, using a combination of ion imaging and U–Pb dating by SIMS, have identified Pb nanoclusters within zircon^12,16,18,22^. They have also been identified using atom-probe tomography (APT), including zircon from Jack Hills in Western Australia^23,24^, in the Napier Complex of East Antarctica^15^, as well as in discordant zircon from ultra-high pressure (UHP) metapelite from the Rhodope Metamorphic Complex in Greece^14^. These nanoclusters are accompanied by either an increased concentration of Y^23^, U^14^, Al^15^ or rare earth elements (REEs)^24^. Furthermore, although Pb nanoclusters have been recorded from both normally discordant and reversely discordant zircon in UHT/UHP rocks, and in concordant zircon from Jack Hills^16^, metallic Pb nanospheres have only previously been reported from reversely discordant zircon in UHT rocks from Antarctica and India.

Any mobility of Pb* will likely affect the ^207^Pb/^206^Pb ratio that is used to calculate the age of a zircon crystal because Pb* is separated from the U from which it was produced, and its isotopic evolution will be enhanced or retarded, depending on the U content in the vicinity of the site to which it migrates. Retarded evolution results in an elevated ^207^Pb/^206^Pb ratio and hence an overestimate of age. As the W74 site at Jack Hills is the location of the world’s oldest known zircon crystals^23,25^, it is imperative to identify any factors that might affect the validity of the measured age of these ancient crystals. Indeed, early studies at this locality, and on zircon from a nearby 3.3 Ga granite, did identify nanometre-scale variations in Pb content within amorphous domains or fission tracks, which were interpreted as Pb* located within the zircon structure and resulting from diffusion through amorphous domains caused by radiation damage^26,27^. A subsequent atom probe study determined that Pb nanoclusters in one of the oldest grains from Jack Hills^28^ were formed during a later thermal event that affected previously radiation-damaged zircon^23^. The nanoclusters were distributed ca. 10–50 nm apart, indicating that they would not result in spurious ages when analyzed by SIMS. However, a later SIMS study^16^ identified a zircon from the W74 site with a concordant ^207^Pb/^206^Pb age of 4463 Ma, which would have made it the oldest known terrestrial zircon. However, careful study confirmed it was located on a local ‘hot spot’ with enhanced clustering of Pb* over an area ~ 20 μm in diameter, similar in size to the SIMS analytical spot that was placed over it during analysis. The true crystallization age of the zircon was estimated as 4300 Ma (million years), thereby establishing that ages can be overestimated by up to ~ 200 Ma as a result of the localized presence of Pb nanoclusters, even in concordant grains. The age discrepancy is even larger (up to 800 Ma) if the ^207^Pb/^206^Pb age is calculated for small individual nanoclusters ~ 5 μm in size using the scanning ion imaging dating technique ^12,16,18^. Because Pb nanospheres contain many more atoms of Pb* per unit volume than Pb nanoclusters (see Supplementary Data), they have an even greater potential to compromise U–Pb age determinations if they were to become locally concentrated.

The present study was, therefore, designed to test a number of these outstanding issues. We selected Hadean and Archean zircon from the W74 site that showed a progression from pristine to more disturbed isotopic systems, including concordant, discordant and reversely discordant grains with a range of δ^18^O and U and OH contents, and classified as pristine (*Group 1*) to progressively more disturbed grains (*Groups 2–5*; as reported in an earlier study^29^). The aims were to see if Pb* distribution could be correlated with the degree of discordance and hence alteration, if it was dependent on variations in the geochemical parameters mentioned above, and, of course, whether metallic Pb nanospheres were present.

## Results

**Figure 1:**
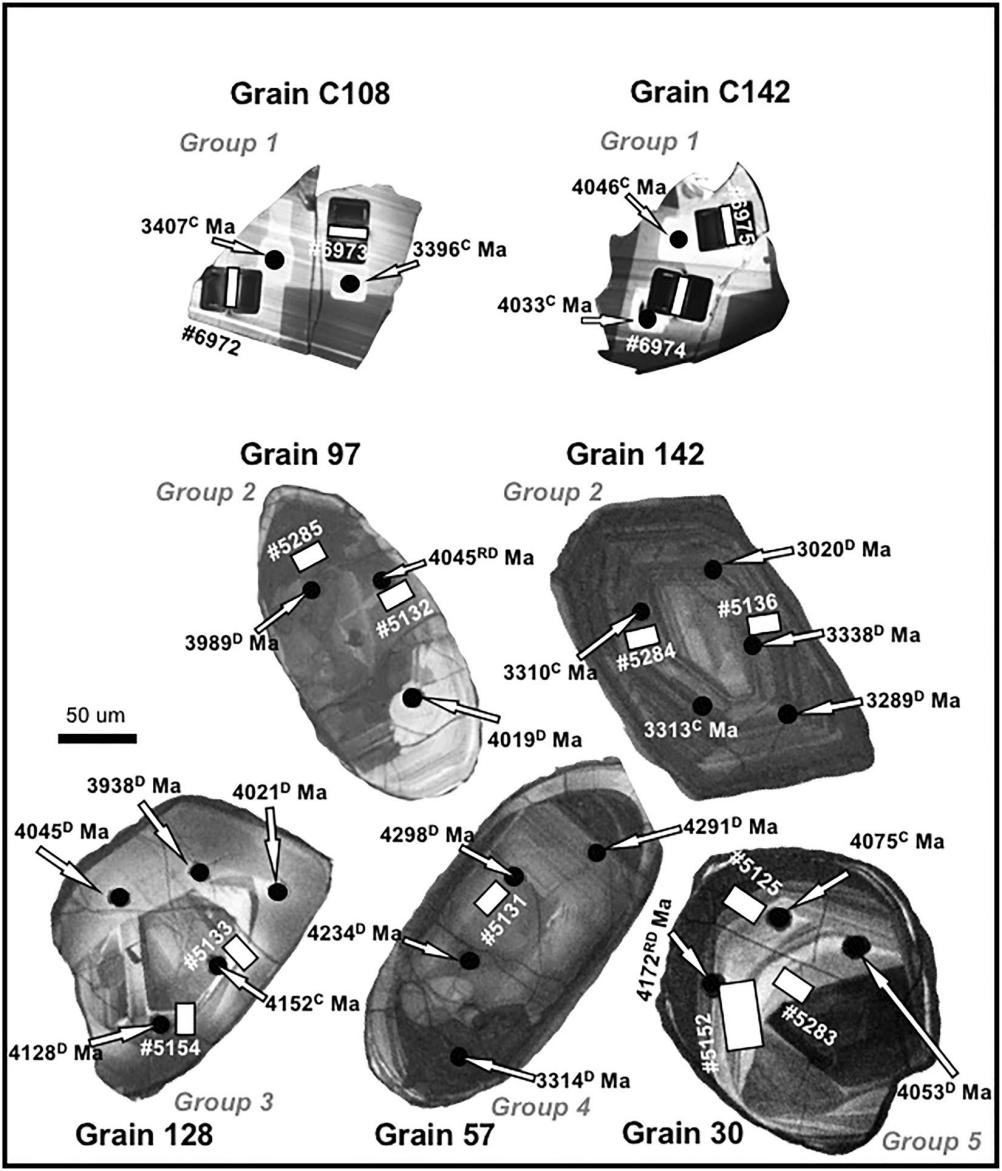
Cathodoluminescence (CL) images of Jack Hills detrital zircon grains from the W74 site used in this study. They are identified by grain and group number (see Table 1 and reference^29^ for further details). Also shown are the U–Pb ages in million years (Ma) of the SIMS sites (black spots) and where the FIB foils were cut (the latter shown as white rectangles, with foil identifying numbers). The letters in superscript after the U–Pb ages are: *C *concordant, *D* discordant, *RD* reversely discordant. Grains C108 and C142 from *Group 1* show additional damage around the FIB sites compared to the other images because they were taken post-TEM analysis.

**Figure 2:**
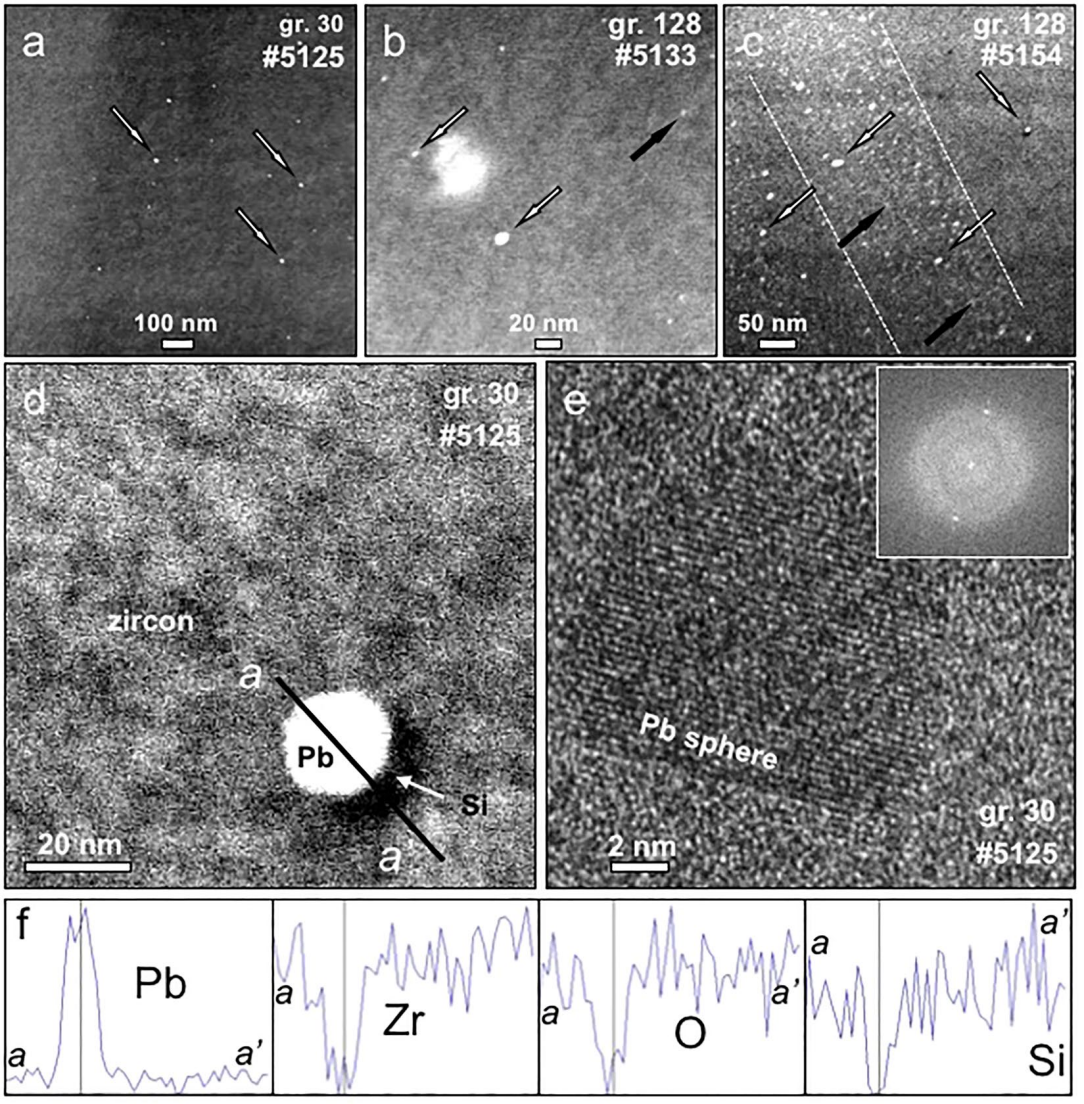
Images showing the distribution and nature of the lead (Pb) nanospheres in zircons of *Groups 3* and *5* from Jack Hills where they are most abundant. (**a**) Random distribution of Pb nanospheres (white spots, with some marked by white arrows) in HAADF image of foil #5125 from grain 30 of *Group 5*; (**b**) HAADF image of foil #5133 showing scattered Pb nanospheres and Y nanoclusters (grey spots, with one marked by a black arrow) in grain 128 from *Group 3*; (**c**) HAADF image of foil #5154 from grain 128 of *Group 3* showing a 150 nm-wide zone of Y enrichment (within white dashed lines). Note that Pb nanospheres are also present, but they are randomly distributed through the image and not concentrated in the Y-rich zone; Pb nanospheres—white arrows: Y nanoclusters—black arrows; (**d**) enlarged HAADF image of foil #5125 from grain 30 of *Group 5* showing detail of Pb nanosphere with partial Si-rich rim (black); black line a–a^1^ marks the profiles shown in (**f**); (**e**) HREM image of foil #5125 from grain 30 of *Group 5* showing Pb nanosphere with diffraction pattern (inset top right corner) confirming cubic symmetry; (**f**) EDS spectra of Pb, Zr, O and Si along profile a–a^’^ in (**d**).

Utilizing transmission electron microscopy (TEM), we analyzed fourteen focused ion beam (FIB) foils cut from seven zircon grains from the W74 site at Jack Hills that exhibited a range in geochemical characteristics^29^ (Fig. 1, Table 1). The foils were cut as close as practical to the SIMS sites and including the same CL domains (Fig. 1). Significantly, eight of the fourteen foils contain Pb nanospheres. These appear as bright spots with well-defined boundaries in high-angle annular dark-field (HAADF) images (Fig. 2a–d) and they are randomly distributed throughout the crystals. Nanospheres are present in grains from *Groups 1 and 3–5* that are either Hadean or Eoarchean in age, making this the first known occurrence of Pb nanospheres in Hadean zircon. However, they are absent in *Group 2* zircon and also in the ca. 3.3–3.4 Ga Paleoarchean zircons. The Pb nanospheres mostly range from 2 to 7 nm in diameter, with a few larger ones up to 20 nm in *Group 3* zircon (Fig. 2d). High resolution imaging (HREM), together with diffraction patterns defining cubic symmetry (Fig. 2e,f and Supplementary Fig. 1), establishes their crystallinity and confirms they are not nanoclusters of disassociated Pb atoms, but are crystals of metallic Pb. Most nanospheres are isolated and not associated with any co-existing phase, although an amorphous Si-rich phase is present adjacent to some nanospheres in *Group 3* (Fig. 2d). In grains from *Group 5*, Y concentrations occur in distinct zones that are also enriched in Pb nanospheres, but unlike the nanoclusters identified from Jack Hills by atom probe tomography^23^, they are not intimately associated with the Pb*, instead forming individual, discrete nanoclusters/nanospheres (Fig. 2c). Importantly, the Pb nanospheres occur in concordant, discordant and reversely discordant grains with SIMS ^207^Pb/^206^Pb ages ranging from 4298 to 3938 Ma, with extremely variable U (40–858 ppm), Th (23–4532 ppm) and water (0.51 to 5.77 of ^16^H^1^O/^16^O × 10^–3^) contents, and with δ^18^O values from 3.93 to 8.93‰ (Table 1). Hence, their occurrence is not controlled by these particular geochemical parameters.

**Table 1:** Results of U–Th–Pb analyses, together with O–OH data and alpha-dose calculations.

Grain/spot	#Foil	^207^Pb/^206^Pb	±σ		[U] (ppm)	[Th] (ppm)	^206^Pb/^204^Pb measured	f_206_%	^16^O cps (× 10^9^)	δ^18^O SMOW‰	±σ	^16^O1H/^16^O (× 10^−^3)	±σ	α-dose# D 10-15 α/mg	±2σ
Group 1							Mount 1								
** C108-1**	6972	**3396**	7	C	57	81	14.970	0.12	0.87	5.80	0.35	0.552	0.21	0.31	0.05
** C108-2**	6973	**3407**	6	C	64	97	171.325	0.01	0.885	5.72	0.34	0.560	0.23	0.35	0.05
** C142-1**	6974	**4033**	11	C	40	23	50.059	0.04	0.881	5.57	0.32	0.522	0.20	0.18	0.03
** C142-2**	6975	**4046**	9	C	46	25	50.669	0.04	0.892	5.26	0.31	0.509	0.25	0.21	0.03
Group 2							Mount B								
97-1	5285	**3989**	3	D	422	336	4250.0	0.44	0.944	5.22	0.34	1.622	0.18	2.03	0.31
97-2	5132	**4045**	2	RD	307	207	48.83	0.04	0.936	5.76	0.37	1.877	0.18	1.45	0.22
142-3	5136	**3338**	5	D	371	2145	2598	0.72	0.970	5.35	0.34	2.109	0.27	3.44	0.52
142-4	5284	**3310**	3	C	369	211	8484	0.22	0.976	5.29	0.35	1.824	0.33	1.71	0.26
Group 3															
128-2	5133	**4152**	5	C	116	43	83.57	0.02	0.957	4.72	0.34	1.650	0.29	0.52	0.08
128-4	5154	**3938**	7	D	858	4532	571.00	3.28	0.929	3.93	0.36	2.010	0.36	7.58	1.14
Group 4															
57-3	5131	**4298**	4	D	529	750	781.00	2.39	0.983	5.77	0.35	3.520	1.42	2.85	0.43
Group 5															
30-2	5283	**4053**	21	D	99	54	87.229	0.02	0.991	6.26	0.34	1.760	0.04	0.45	0.07
30-3	5125	**4075**	16	C	59	36	79.92	0.02	0.988	6.57	0.33	1.710	0.37	0.27	0.04
30-4	5152	**4172**	11	RD	56	30	403.74	0.00	0.988	8.93	0.34	5.770	2.17	0.26	0.04

Data and grain numbers are from reference^29^. The Pb nanospheres are present in all Hadean and Eoarchean zircon grains, except for Group 2.

*f*_*206*_*% *the amount of common ^206^Pb, estimated from measured ^204^Pb, ^*207*^*Pb/*^*206*^*Pb* age given in million years, *C *concordant, *D* discordant, *RD *reversely discordant, *SMOW *standard mean ocean water.

Bold font indicates foils containing Pb* nanospheres.

In detail, zircon grains from *Group 1* are pristine, concordant and have no cracks or inclusions, but only the Hadean crystal (grain C142) has Pb nanospheres. In *Group 2*, although some grains show considerable discordance^29^, others are pristine (Fig. 2d) yet no nanospheres were documented in either the Hadean or the Paleoarchean crystals.

**Figure 3:**
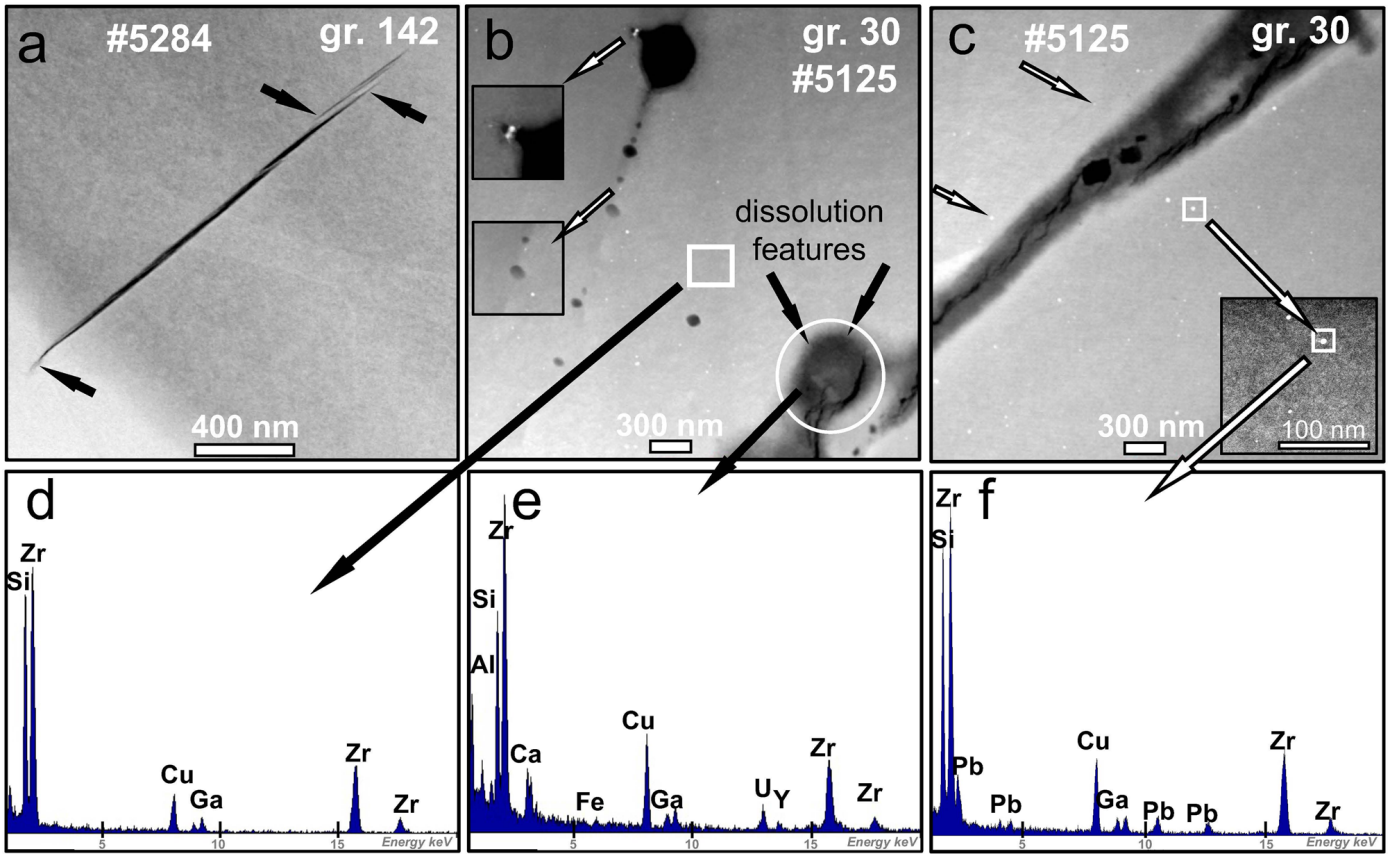
HAADF Z-contrast TEM images of zircon containing cracks and pores. (**a**) Example of sharply defined crack in foil #5284 from grain 142 of *Group 2*; note the pointed terminations marked by black arrows. There are no Pb nanospheres in this group; (**b**) pores (grey/black) along trails of healed cracks in foil #5125 from grain 30 of *Group 5*. White arrows pointing to insets highlight relationship between Pb nanospheres and the healed crack and pores in the top-left of the image; dissolution features are evident around the pore in the bottom right corner of the image (within white circle). The white square and white circle refer to panels (**d,e)**, respectively; (**c**) in the same foil as (**b**), a healed crack with blurred margin contains pores (black) and is in close proximity to Pb nanospheres (two identified by small white arrows). Large white arrows identify a Pb nanosphere that is enlarged in the inset and linked to the image in (**f)**; (**d–f**) EDS spectra of areas marked in (**b,c)** and selected to show the composition of (**d**) an area of zircon matrix; (**e**) a crack with a pore; and (**f**) a Pb nanosphere. Note that Cu comes from the copper grid where the foil is placed and Ga gets implanted during the foil-cutting process: they are therefore artifacts and do not come from the sample.

We identified the presence of cracks and pores in all FIB foils taken from zircon grains in *Groups 2–5*, and, although they are not abundant, we investigated these further in order to test if there was any association between Pb* and subsequent alteration. Cracks with sharp edges and tips are present in *Group 2* zircon (Fig. 3a), but no Pb nanospheres were documented in these grains. Grains from *Groups 3–5* do contain nanospheres, but here the cracks have blurred interfaces and dissolution features (Fig. 3b,c), locally associated with pores. Careful examination revealed only one example of zircon (from *Group 5,* Fig. 3b) where a trail of fluid inclusions marking the site of a former crack also contains a single nanosphere. Energy dispersive spectrometry (EDS) analyses indicate enrichment of Y, U, Ca, K, Al and Fe in the crack (Fig. 3e; Supplementary Data Fig. S2). Importantly, these elements were not detected in the adjacent host zircon (Fig. 3f). Hence, although the cracks form pathways for fluid penetration, their annealing post-dates nanosphere formation and any association appears random. Thus, Pb nanospheres, once formed, remain stable during dissolution–re-precipitation processes and are unaffected by further fluid infiltration. As with the cracks, pores occur in some zircon grains (Fig. 3b,c) but are also independent of Pb nanosphere distribution: there is thus no connection between Pb nanospheres and the development of pores or dislocations. In summary, nanospheres are present only in Hadean and Eoarchean zircons from Jack Hills and occur independent of the degree of alteration or whether the grains are concordant, discordant or reversely discordant.

## Discussion

The fundamental questions posed by the mobility of radiogenic lead in zircon are why and how nanoclusters and nanospheres form, and the relationship between them. Later deformation and the generation of dislocations was favoured by some authors^15^ for the formation of Pb nanoclusters, whereas Pb migration into dislocation ‘loops’ formed by annealing of radiation damage has also been proposed^14^. The regular distribution of nanoclusters at 10–50 nm intervals in Hadean zircon from Jack Hills suggested that movement was limited and likely the result of local mobilization of Pb* atoms initially formed by alpha decay of U and Th^23^, This was supported by the later study of a low U Hadean zircon where Pb* concentrations corresponded to differences in CL patterns, suggesting they formed in zones originally more enriched in radioactive elements^16^, although overall, the crystal recorded U contents of < 50 ppm, and with no evidence of metamorphism, deformation, or extensive recrystallisation. We validate these observations here and further identify the presence of metallic Pb nanospheres in Jack Hills zircon that has excellent crystallinity and a lack of mosaic structure, as determined by TEM (Supplementary Fig. 1a); hence no evidence of extensive metamict alteration as a precursor to nanosphere formation.

In zircon from UHT rocks of the Napier Complex in East Antarctica^20^ that contain Pb nanospheres, radioactive elements were sufficiently abundant to result in extensive amorphous, metamict domains. However, islands of crystallinity remained and it was argued that temperatures > 900 °C concentrated Pb* into melt inclusions, together with Ti, Al and Si^20^. Upon annealing, a mosaic of micro-crystals was formed with slightly misoriented lattices within the damaged zircon structure, thus preserving the nanospheres. However, this cannot explain the occurrence of Pb nanospheres in Jack Hills zircon as there is no evidence they ever attained UHT conditions. Titanium-in-zircon thermometry indicates most Jack Hills zircon records crystallization temperatures of 680–720 °C ^30,31^, and no zircon recording a temperature > 900 °C has ever been reported from Jack Hills. In the earliest study of Ti-in-zircon applied to UHT rocks^32^, Ti was considered to be immobile, whilst it was further noted that the recorded temperatures were below those expected for UHT rocks; a feature noted in several subsequent studies^33,34^. However, the applicability of Ti-in-zircon thermometry to rocks and minerals that have undergone high-grade metamorphism has been widely investigated over the past decade and, although both Pb and Ti have low thermal diffusivity under most crustal conditions^35^, high temperature experimental studies have revealed enhanced diffusion of Ti along open channels parallel to the zircon c-axis, although there was minimal mobility at temperatures < 800 °C^36^. Furthermore, Ti was mobilised along with Pb in the original study where Pb* nanospheres were identified in orthogneisses from the Tula Mountains in Antarctica^20^, hence diffusion has the potential to modify the original Ti-in-zircon temperature in pre-existing igneous zircon. A study of UHT rocks from the Napier Complex of Antarctica (including sites from which Pb nanospheres were discovered), has confirmed that igneous Ti-in-zircon temperatures can be modified and, although both magmatic and metamorphic zircon cores recorded lower temperatures than newly-generated rims, they both had Ti-in-zircon values consistent with UHT conditions^33^. Therefore, once attained under UHT conditions, the temperature recorded by the Ti geothermometer is robust and remains unaffected by later retrogression^33,34^. The only way to modify the temperature would be to metamorphose the zircon under even higher UHT conditions. Based on these studies, it is evident that if Jack Hills zircon had ever attained such temperatures, then these would still be recorded by the grains; but this is not the case^30,31^. Hence, this implies that Pb nanospheres can form at temperatures lower than those of UHT metamorphism and, as such, opens up the possibility they may be more widely distributed in zircon than previously recognized.

Another finding from this study is that Pb nanospheres are present in zircon irrespective of the degree of later disturbance. In both the Antarctic and Indian examples, metallic Pb nanospheres were identified in reversely discordant zircon, indicating the analysed crystal domains had essentially gained radiogenic lead, with this leading to dates that were spuriously older than the actual crystallization age of the zircon. Therefore, this is the first known case where Pb* nanospheres are present in zircon grains that record concordant U–Pb ages (Table 1).

Unlike Pb nanoclusters, where atoms can readily diffuse if zircon is subjected to an increase in temperature, pressure or deformation, the formation of Pb nanospheres effectively immobilises Pb*, freezing the ^207^Pb/^206^Pb ratio and preventing further redistribution of the lead and its participation in later reactions. This is partly because of the lead occurring as dense, cubic nanocrystals, which means they have high surface energy per unit volume, with confining pressure on the nanosphere far exceeding that on the zircon host^37^. This concentration of Pb* in nanospheres potentially has implications for geochronology, as it means that any zircon containing such nanospheres will now contain Pb* in two discrete states; Pb* ‘frozen’ in the metallic nanospheres with a ^207^Pb/^206^Pb ratio fixed at their time of formation, and additional Pb* that formed from post-nanosphere breakdown of U and Th in adjacent zircon domains and having its own distinct ^207^Pb/^206^Pb ratio based on time elapsed to the present day. This is also true for Pb clusters and nanoclusters, where their ^207^Pb/^206^Pb ratio will record the time of formation^23,24^. Such a scenario would potentially have a detrimental effect on obtaining a precise U–Pb age where Pb nanoclusters and nanospheres are sufficiently large or numerous, making-up a significant proportion of the Pb* balance at the analytical site, or if the analytical spot size, and hence volume, is extremely small, as previously shown for Pb nanoclusters^16^.

As a more positive consequence of Pb nanosphere formation, their individual ^207^Pb/^206^Pb ratios can be determined by atom probe tomography or, if they are sufficiently large, by NanoSIMS. In a study of Antarctic zircon^38^, the ^207^Pb/^206^Pb ratios of individual Pb nanospheres were determined by NanoSIMS, generating a Nanosphere Model Age (NMA) of ~ 2610 Ma and a ~ 3110 Ma age for zircon crystallization. The former is consistent, within error, with the timing of UHT metamorphism in the Napier Complex, whereas the latter is consistent with igneous zircon ages previously obtained from the Napier Complex^19^.

Regarding the time and method of formation of Pb nanoclusters at Jack Hills, the 4374 Ma detrital igneous zircon examined by atom probe tomography^23^ had a thin but distinct rim. The ^207^Pb/^206^Pb age of that rim was ~ 3400 Ma, with a Th/U ratio of 1.20^24^. The age is similar to both granitoids adjacent to the Jack Hills belt^39^ and abundant detrital igneous zircon in sediments throughout the belt^40^, suggesting that the rim was magmatic in origin. By taking atom probe measurements of the ^207^Pb/^206^Pb ratio both within and outside the nanoclusters, this enabled calculation of the time elapsed between formation of the zircon and the nanoclusters, revealing that redistribution of Pb* (and associated Y) occurred at ~ 3.4 Ga, the age of the rim-forming event. Unfortunately, of the grains chosen for the present investigation, only one (grain 57 from *Group 4*; Fig. 1) contains a rim wide enough to analyze, and this was not determined as part of the earlier study^29^. However, a highly-discordant, U- and Th-rich area from an altered domain in close proximity to the rim recorded a very imprecise ^207^Pb/^206^Pb date of 3314 ± 296 Ma^29^, but broadly within error of the 3.4 Ga event. Rims with ages of 3300–3600 Ma are common in the detrital zircon population at the W74 and adjacent sites at Jack Hills^28,40^, so magmatic events at this time may well have affected the zircon grains utilized in this study. However, the small size and scattered distribution of the nanospheres in Jack Hills zircon would make the calculation of their ^207^Pb/^206^Pb ratios difficult, even by atom probe, and so the timing of Pb nanosphere formation has not been precisely constrained. Importantly, none of the 3.3–3.4 Ga grains in this study contained Pb nanospheres, even though their U and Th contents are quite high in *Group 2* grains of this age (Table 1). This raises three possible scenarios: (i) the nanospheres formed during one or several events that took place in the Hadean/Eoarchean, (ii) they were formed at some time between 3.9 Ga (youngest zircon containing nanospheres) and 3.4 Ga, or (iii) they formed during the 3.4 Ga event identified in the area, possibly indicating the grains were originally xenocrysts derived from the magmatic protolith of the Paleoarchean granitoids. It is certainly evident that they formed before the cracks and pores and also the ‘zero’ Ma age recorded by the lower intercepts of the U–Pb data^29^. As previously noted, this establishes that once formed, Pb* nanospheres are not affected by subsequent alteration.

Notwithstanding the above scenarios concerning their time of origin, it needs to be considered if later metamorphism affected the Pb nanosphere-bearing zircon crystals, as this was shown to be the case with the Antarctic zircons^20^. Although Pb* can migrate at low temperature if zircon grains have undergone radiation damage, the metamorphic conditions at the W74 site at Jack Hills only reached upper greenschist facies, with temperatures estimated from monazite-xenotime thermometry at 420–475 °C ^41^, based on both inclusions in zircon and grains in the quartz-muscovite matrix of the host conglomerate. Time constraints were placed on this event by the dating of metamorphic monazite from the host conglomerate, which recorded an age of 2653 ± 5 Ma^42^. Metamorphic xenotime inclusions in zircon also recorded a similar, but less precise age of ~ 2680 Ma ^41^. In addition, a radiation damage age of 1120 Ma was reported for zircon from the W74 site^43^, estimated to be the time of latest annealing of the zircon structure. Based on a Pb nanosphere being present within an annealed crack in *Group 3* zircon (Fig. 3b), this event must have post-dated nanosphere formation, which was therefore earlier than ~ 1100 Ma. It is also highly unlikely that greenschist facies conditions were sufficient to mobilize Pb* formed by the decay of U and Th and residing in trails of alpha damage, although such a process cannot be totally dismissed as having the potential to drive initial clustering. It is therefore considered that the Pb nanospheres were formed before the 2.6 Ga greenschist facies metamorphic event. The other theoretical possibility, as noted above, is that the Jack Hills Hadean/Eoarchean zircons were metamorphosed under UHT conditions prior to being affected by the 3.4 Ga magmatic event and being incorporated in the host conglomerate. However, given that Pb and Ti are intimately associated in Antarctic zircon with both nanoclusters and nanospheres^18,20^, and are clearly immobilized by this process, it would be expected that any grain affected by UHT metamorphism would record Ti-in-zircon temperatures in accord with UHT conditions, but, as discussed above, this is not supported by the evidence^30,31^. So, unlike the Antarctic^20^ and Indian^21^ examples, UHT metamorphism is shown not to be a prerequisite for nanosphere formation at Jack Hills.

The other fundamental question raised was whether there is a relationship between Pb nanoclusters and nanospheres. Two possible scenarios for Pb clustering in Jack Hills zircon have been proposed, both based on the initial distribution of Pb* atoms formed by alpha-decay of U and Th: (1) as a result of re-mobilization during a major thermal event^23^ and (2) concentration in zones that had higher initial U and Th contents, but likewise triggered by a later thermal event^16^. In the case of Pb nanospheres in both Antarctica^20^ and India^21^ they were interpreted to have formed during UHT metamorphism by remobilisation of Pb* in metamict domains that resulted from large-scale alpha-decay in U- and Th-rich zircon. But from our study of Jack Hills zircon, Pb nanospheres formed without evidence of ever undergoing UHT metamorphism. Nonetheless, combined U–Pb dating and Hf-in-zircon isotopic studies^40,44^ establish that the granitoids marginal to the Jack Hills belt evolved though progressive reworking of Hadean crust, with only limited addition of new mantle material. Whereas the bulk of the Paleo- to Neoarchean zircon in sediments throughout the belt are attributed to reworking of these granitoids^40^, there is no known source for the Hadean gains. Such a long and complex history prior to inclusion in the host conglomerate makes it likely that the Hadean/Eoarchean zircons were affected by multiple thermal events over a considerable period of time, with each event having the potential to mobilise radiogenic lead. Because nanospheres contain many more Pb* atoms than nanoclusters (see Supplementary data), it is attractive to suggest that progressive mobilisation could lead to Ostwald ripening of Pb* in the nanoclusters^20^, with these representing a precursor stage and, therefore, a pre-requisite for Pb nanosphere formation. We were unable to test this during the present study, because TEM analysis is unable to resolve single atoms, even if they are clustered.

Finally, as yet, there is no evidence that the presence of nanospheres has adversely affected U–Pb dating of zircon, because their volume is small compared to that of the average SIMS analytical spot. However, although this was also the conclusion of the initial ATP study of Pb nanoclusters in Jack Hills zircon^23^, a later SIMS study showed they had a profound effect^22^. With ~ 280× more atoms per unit volume than nanoclusters (Supplementary Data), the effect of Pb nanospheres would be far more dramatic. When coupled with the broad desire in the analytical community to measure ever-smaller volumes of material, researchers need to be aware of this potential problem and take steps to identify situations where there is the possibility that Pb nanoclusters and nanospheres might be present. As noted in numerous previous studies^12,17,18,45^, irregular sputtering of ^206^Pb and ^207^Pb during SIMS analysis provides direct evidence that unsupported Pb* is present at the analytical site and thus provides a red flag that further investigation is required.

## Methods

### Strategy and site selection

Fourteen foils were prepared from 7 Jack Hills zircon grains from the W74 site^25^. The grains had previously been characterized in an earlier study^29^ (Fig. 1, Table 1), where the authors separated the zircons into five groups (*Groups 1–5*) based on increasing degrees of disturbance. *Group 1* contained only concordant grains with no significant amount of common Pb, and all recorded mantle δ^18^O values. *Group 2* contained grains with both concordant and discordant domains, with OH at or above background, but with relatively undisturbed mantle δ^18^O values. *Group 3* zircons recorded complex U–Pb systematics, with both concordant and discordant domains, together with low δ^18^O and OH values similar to *Group 2*. Zircon grains from *Group 4* had increased values of δ^18^O and OH, and defined a discordia with a lower intercept close to zero million years. *Group 5* consisted of complex zircons with U–Pb systematics that did not fit the simple discordance pattern found in several of the previously described groups. For this study, two *Group 1* grains were analysed, grain C108 and grain C142 (2 foils from each grain); two grains from Group 2, grains 97 and 142 (note that this is different from grain C142 of *Group 1*) (2 foils from each grain); grain 128 from *Group 3* (2 foils), grain 57 from *Group 4* (1 foil) and grain 30 from *Group 5* (3 foils) (Fig. 1).

The site-specific focused-ion-beam (FIB) technique was used to prepare foils for transmission electron microscopy (TEM). This method involves cutting an electron-transparent foil from pre-selected areas of interest^46–48^. The TEM foils are 15–20 μm wide, 10–15 μm deep and 100 nm thick. A glass fibre attachment to a micro-manipulator was used to lift out the foils: details of the technique are given elsewhere^48,49^.

The FIB foil sites were selected as close as possible to the U–Pb analytical sites utilised in the previous study^29^ and including the same structural domain as determined using cathodoluminescence (CL) images (Fig. 1). It was not possible to traverse the actual U–Pb sites, due to earlier damage caused during collection of the age data. The site selection was made to include concordant, discordant and reversely discordant domains (Table 1) of various ages and U, oxygen and water contents. All grain numbers and analytical data are the same as in reference^29^. Table 1 contains α-dose calculations, calculated at 1120 Ma, the time the grains were last annealed^38^. Zircon grains C106 and C142 from *Group 1* were selected to examine pristine grains from both the Hadean and Paleoarchean populations. Grain C106 records a narrow age range from 3396 ± 7 to 3407 ± 6 Ma, with relatively low U and Th contents and δ^18^O from 5.72 to 5.80‰ (Table 1). Grain C142 also records a uniform age, ranging from 4033 ± 11 to 4046 ± 9 Ma, with low U and Th contents and δ^18^O from 5.26–5.57 ‰ (Table 1). Zircon grains 97 and 142 from *Group 2* have relatively uniform oxygen, U and Th contents, with one extremely high Th (> 2000 ppm) value, and recorded a range of U–Pb ages from Hadean to Paleoarchean (Table 1). The two foils obtained from grain 97 were taken from domains with a range in U content from 307 to 422 ppm and in δ^18^O from 5.22 to 5.76 ‰ (Table 1). The two foils from grain 142 were from domains with 369–371 ppm of U and δ^18^O of 5.22–5.76‰ The ages of the selected areas in grain 97 were 3989 ± 3 Ma and 4045 ± 2 Ma and in grain 142 they were 3338 ± 5 Ma and 3310 ± 3 Ma (Fig. 1). The two FIB foils cut from *Group 3* zircon grain 128 were from domains with very different U and Th contents. One site (128-2) with a concordant age of 4152 ± 5 Ma had 116 ppm U and 43 ppm Th. The other site (128-4) recorded a discordant age of 3938 ± 7 Ma and had 838 ppm U and 4532 ppm Th. The domain from which the single FIB foil was taken from grain 57 of *Group 4* had 529 ppm U and a δ^18^O value of 5.77‰. The analysed area was selected from a discordant site (57-3) that recorded the oldest age (4298 ± 4 Ma) of all grains analysed in this study. Grain 30 from *Group 5* recorded very low U (maximum 99 ppm) and Th (maximum 54 ppm) contents (Table 1). One FIB section was cut from a discordant part of the zircon (30-2) with an age of 4053 ± 21 Ma, another from a concordant area (30-3) with an age of 4075 ± 17 Ma (both with low ^16^H^1^O/^16^O × 10^–3^ contents of 1.76 and 1.71, respectively), and the third from a reversely discordant part (30–4) with an age of 4172 ± 11 Ma. The latter was from an area with a high water content (5.77 ^16^H^1^O/^16^O × 10^–3^). Oxygen values in this grain increased from ~ 6 ‰ near 30-2 to ~ 9‰ near 30-4, with an accompanying rise in OH (Table 1).

### Equipment

Information on the techniques used for determining the U–Pb ages, oxygen values and OH contents of the zircon grains used in this study are presented in reference^29^. For this study, analytical and energy-filtered high-resolution transmission electron microscopy (ATEM, HRTEM) together with high-angle annular dark-field (HAADF) TEM imaging was performed using a FEI Tecnai™ G2 F20 X-Twin at GFZ Potsdam, operated at 200 kV with a field emission gun (FEG) electron source. The TEM is equipped with a post-column Gatan imaging filter (GIF Tridiem). The HRTEM images were energy-filtered using a 10-eV window on the zero-loss peak. Analytical electron microscopy (AEM) was performed with an EDAX X-ray analyser equipped with an ultra-thin window. The X-ray intensities were measured in scanning transmission mode (STEM) where the electron beam is serially scanned over a pre-selected area, thus minimizing mass loss during data acquisition.

## Supplementary Information

Supplementary Information.

## Data Availability

The data sets used and/or analyzed during the current study are available from the corresponding author on reasonable request.
