# Supplementary material for: Metallic lead (Pb) nanospheres discovered in Hadean and Eoarchean zircon crystals at Jack Hills
Source: Sci Rep. 2023 Jan 17;13:895. doi: 10.1038/s41598-023-27843-6 (PMC9845240; doi:10.1038/s41598-023-27843-6)
Supplement: Supplementary file 1 — Supplementary Information. [file 41598_2023_27843_MOESM1_ESM.pdf]

Supplementary Data

Metallic lead (Pb) nanospheres discovered in Hadean and Eoarchean zircon crystals at Jack Hills

Monika A. Kusiak[1], Richard Wirth[2], Simon A. Wilde[3], Robert T. Pidgeon[3]

[1]*Institute of Geophysics, Polish Academy of Science, ul. Księcia Janusza 64, PL-01452 Warsaw, Poland.*

[2]*GeoForschungsZentrum, Potsdam, Telegrafenberg, Section 3.5 Surface Geochemistry D-14473, Potsdam, Germany.*

[3]*School of Earth and Planetary Sciences, Curtin University, PO BOX U1987, WA 6845, Perth, Australia.*

Lead (Pb) atom calculations for nanoclusters and nanospheres

With respect to the calculations used for comparing Pb nanoclusters and Pb nanospheres, the Pb unit cell is 4.9 $Å^3$ or 117.65 $Å^3$ (with 14 atoms). Assuming a 'typical' Pb cluster (taken from reference[1]) of 10 nm, this will contain approximately 4000 Pb atoms. Because they are widely distributed (10-50 nm apart[1]), they would not have any significant effect on U-Pb dating by SIMS, utilizing a ~25 μm spot. However, when concentrated into tighter nanoclusters up to 20 μm[2], they can result in spurious ages at least 200 Ma older than the 'true' age of the zircon.

In contrast, a Pb nanosphere with a radius of 1.5 nm, will contain 120 unit cells and 1681 Pb atoms per nanosphere. But if we take a Pb nanosphere with a radius of 15 nm, this will contain 120,103 unit cells, which gives 1,681,000 Pb atoms per nanosphere. Therefore, even a moderately-sized Pb nanosphere will significance increase the local concentration of Pb*.

Additional Images

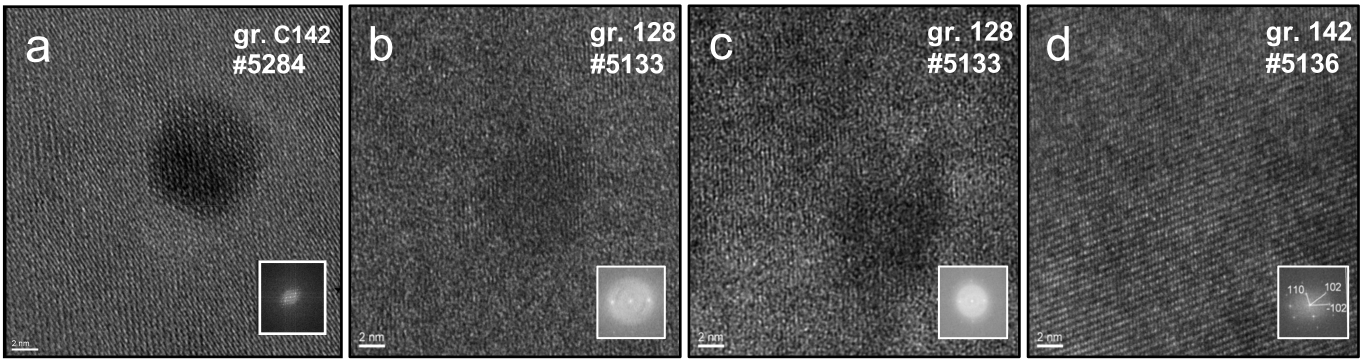

Supplementary Figure 1. TEM HREM lattice fringe images of **a, b, c** Pb nanospheres and **d**, highly crystalline zircon, with grain and foil numbers. Insets in **a, b** and **c** show diffraction patterns (FFT) of the Pb nanospheres, and in **d** it shows excellent crystal structure. Note structure of zircon in **a** (*Group1*) is pristine as in **d** (*Group 2*).

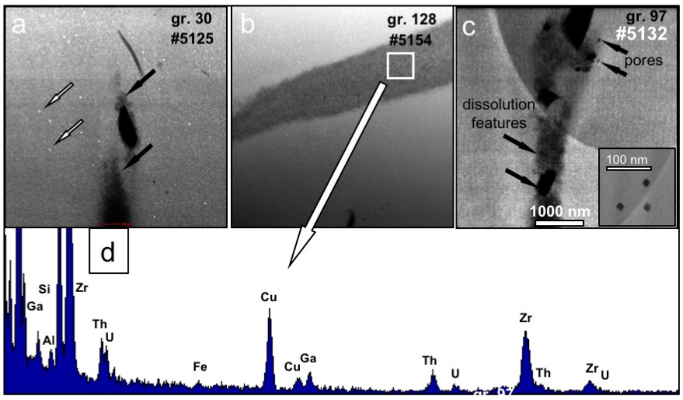

Supplementary Figure 2. HAADF Images showing imperfections in zircon grains with Pb nanospheres. **a**, Pores with dissolution features (marked by black arrows) and with Pb nanospheres outside the pores (white arrows); #5125; **b**, Healed crack, #5154; **c**, crack with dissolution features and fluid inclusions (dark features, several indicated by black arrows). Inset shows three rhombic-shaped inclusions, #5132; **d**, EDS analysis made inside the crack shown in **b** and marked by rectangle. Cu peaks generated from the Cu-grid into which the FIB foil is placed; Ga peaks come from Ga implanted during the FIB foil-cutting process. Both are artefacts and do not reflect the element content of the analyzed sample.

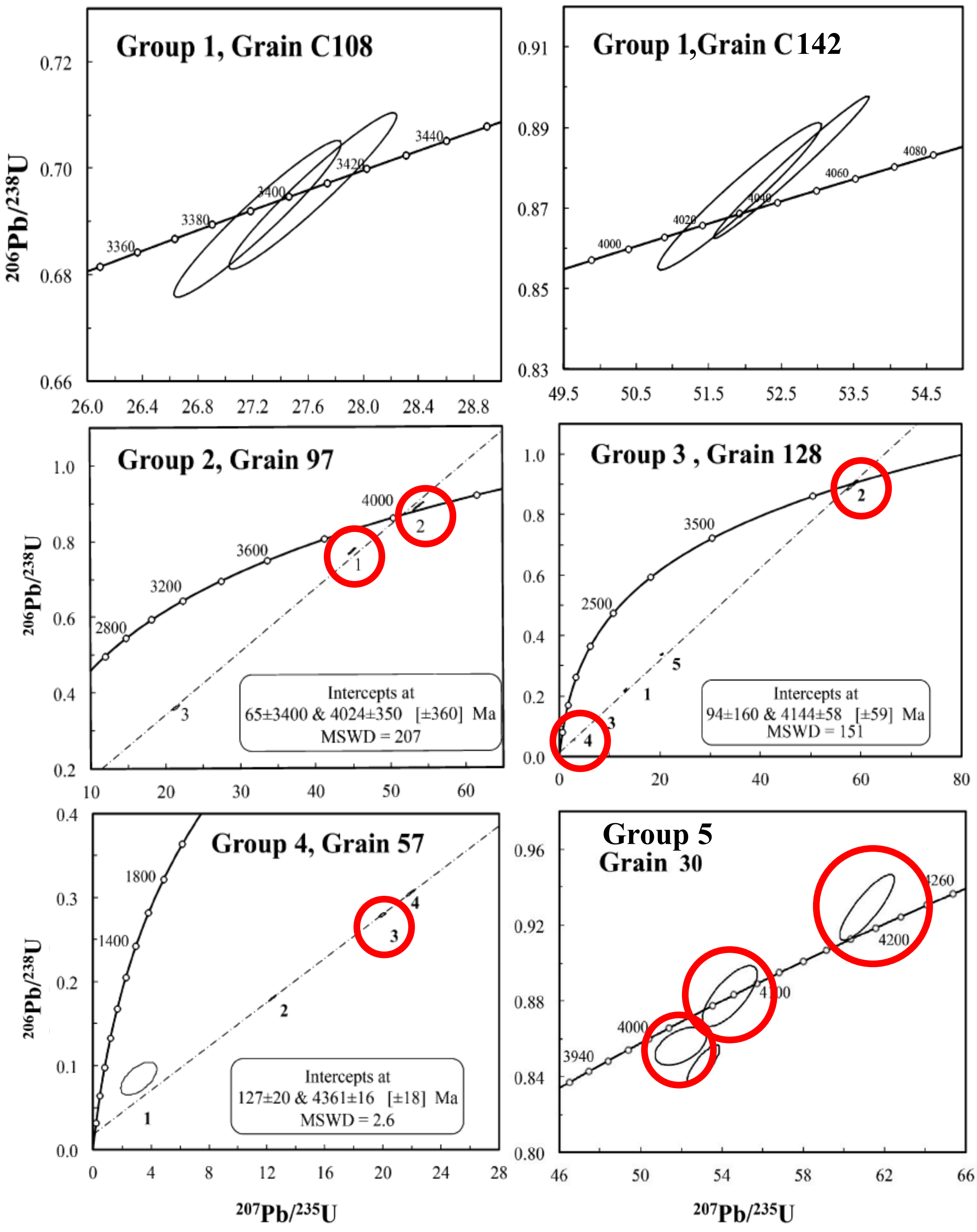

Supplementary Figure 3. U-Pb concordia diagrams for zircons selected for the present study[3]. Red circles – ages of the areas in the grain where the FIBs were cut and the TEM analyses performed. The numbers refer to the data in Table 1 and ref[29] For *Group 1*, all four sites were analyzed.

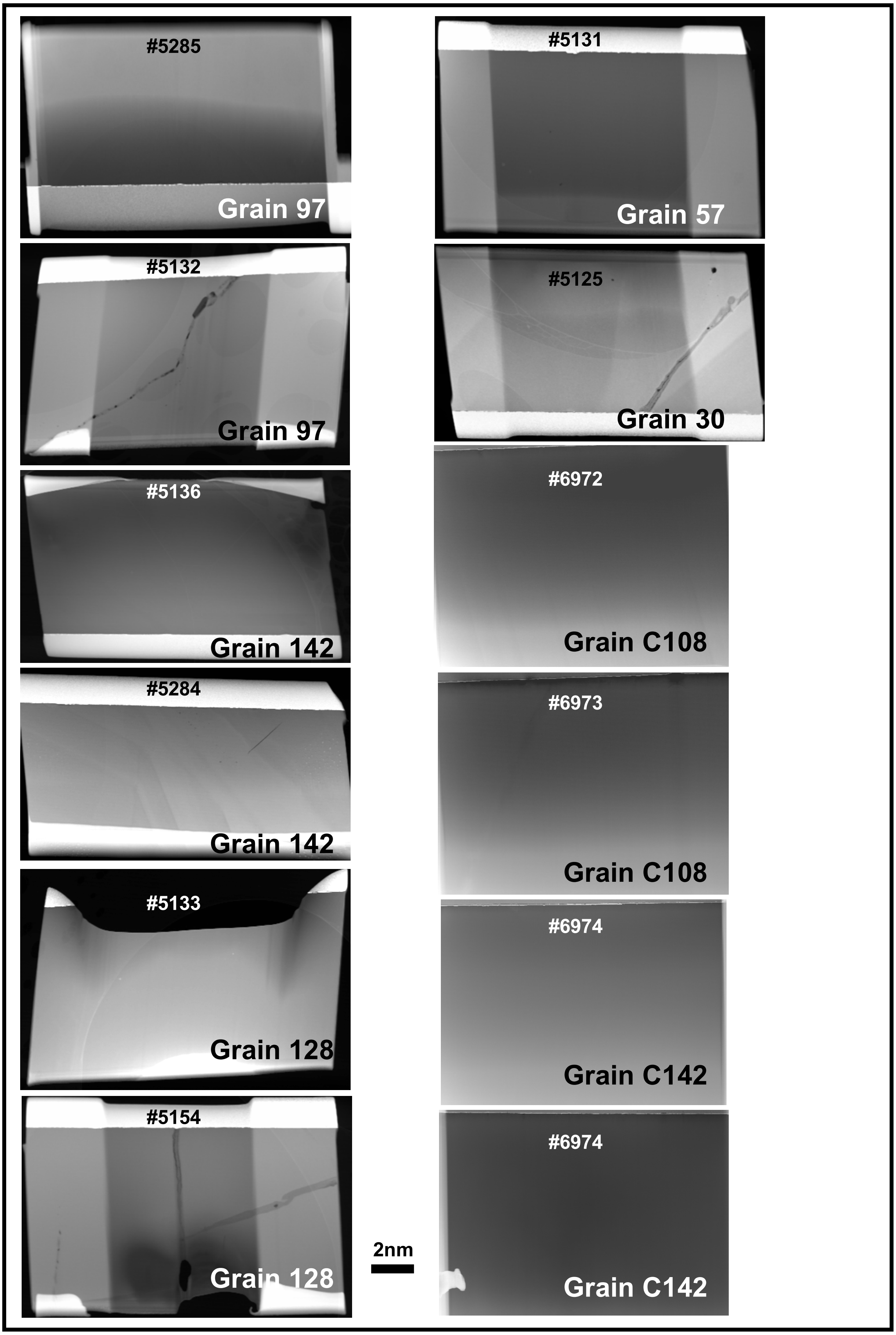
#5285
Grain 97
#5131
Grain 57
#5132
Grain 97
#5125
Grain 30
#5136
Grain 142
#6972
Grain C108
#5284
Grain 142
#6973
Grain C108
#5133
Grain 128
#6974
Grain C142
#5154
Grain 128
#6974
Grain C142
2nm

Supplementary Figure 4. TEM images of focussed-ion beam foils (FIBs) taken from zircon grains analysed in this study. The image of foil #5152 from grain 30 was not retained. Note also that the images of grains C108 and C142 appear different as they were prepared at a later time using updated equipment.

References

1. Valley, J. W. *et al.* Hadean age for a post-magma-ocean zircon confirmed by atom-probe tomography. *Nat. Geosci.* **7**, 219-223 (2014).
2. Ge, R. *et al.* A 4463 Ma apparent zircon age from the Jack Hills (Western Australia) resulting from ancient Pb mobilization. *Geology* **46**, 303-306 (2018).
3. Pidgeon, R. T., Nemchin, A. A. & Whitehouse, M. J. The effect of weathering on U–Th–Pb and oxygen isotope systems of ancient zircons from the Jack Hills, Western Australia. *Geochim. Cosmochim. Acta* **197**, 142-166 (2017).
